# Supplementary material for: COZOID: contact zone identifier for visual analysis of protein-protein interactions
Source: BMC Bioinformatics. 2018 Apr 6;19:125. doi: 10.1186/s12859-018-2113-6 (PMC5889581; doi:10.1186/s12859-018-2113-6)
Supplement: Supplementary file 2 — Software build. Executable binary file of the software tool. (ZIP 118,784 kb) [file 12859_2018_2113_MOESM2_ESM.zip › cozoid/platform/docs/testng-6.5.1-javadoc.zip!/org/testng/xml/XmlSuite.html]

XmlSuite (TestNG)


---


|  |  |  |
| --- | --- | --- |
| **Overview** \| **Package** \| **Class** \| **Tree** \| **Deprecated** \| **Index** \| **Help** | |  |
| **PREV CLASS**   **NEXT CLASS** | **FRAMES**    **NO FRAMES**     **All Classes** |  |
| SUMMARY: NESTED \| FIELD \| CONSTR \| METHOD | DETAIL: FIELD \| CONSTR \| METHOD |  |


---


## org.testng.xml Class XmlSuite

```
java.lang.Object
  org.testng.xml.XmlSuite
```

**All Implemented Interfaces:**: java.io.Serializable, java.lang.Cloneable

---

``` public class XmlSuite extends java.lang.Object implements java.io.Serializable, java.lang.Cloneable ```

This class describes the tag <suite> in testng.xml.

**Author:**
:   Cedric Beust, Alexandru Popescu

**See Also:**: Serialized Form

---

| **Field Summary** | |
| --- | --- |
| `static java.lang.String` | `CONTINUE` |
| `static java.lang.Boolean` | `DEFAULT_ALLOW_RETURN_VALUES` |
| `static java.lang.String` | `DEFAULT_CONFIG_FAILURE_POLICY`             Whether to SKIP or CONTINUE to re-attempt failed configuration methods. |
| `static java.lang.Integer` | `DEFAULT_DATA_PROVIDER_THREAD_COUNT`             Thread count for the data provider pool |
| `static java.lang.Boolean` | `DEFAULT_GROUP_BY_INSTANCES`             By default, a method failing will cause all instances of that class to skip |
| `static java.lang.Boolean` | `DEFAULT_JUNIT`             JUnit compatibility flag. |
| `static java.lang.Boolean` | `DEFAULT_MIXED`             mixed mode flag. |
| `static java.lang.String` | `DEFAULT_PARALLEL` |
| `static java.lang.String` | `DEFAULT_PRESERVE_ORDER` |
| `static java.lang.Boolean` | `DEFAULT_SKIP_FAILED_INVOCATION_COUNTS` |
| `static java.lang.Integer` | `DEFAULT_THREAD_COUNT`             The thread count. |
| `static java.lang.Integer` | `DEFAULT_VERBOSE`             The suite verbose flag. |
| `static java.lang.String` | `PARALLEL_CLASSES` |
| `static java.lang.String` | `PARALLEL_INSTANCES` |
| `static java.lang.String` | `PARALLEL_METHODS` |
| `static java.util.Set<java.lang.String>` | `PARALLEL_MODES` |
| `static java.lang.String` | `PARALLEL_NONE` |
| `static java.lang.String` | `PARALLEL_TESTS`             Parallel modes |
| `static java.lang.String` | `SKIP`             Configuration failure policy options |


**Constructor Summary**

`XmlSuite()`


| **Method Summary** | |
| --- | --- |
| `void` | `addExcludedGroup(java.lang.String g)` |
| `void` | `addIncludedGroup(java.lang.String g)` |
| `void` | `addListener(java.lang.String listener)` |
| `java.lang.Object` | `clone()`              Note that this is not a full clone: XmlTest children are not cloned by this method. |
| `boolean` | `equals(java.lang.Object obj)` |
| `java.lang.Boolean` | `getAllowReturnValues()` |
| `java.util.Map<java.lang.String,java.lang.String>` | `getAllParameters()` |
| `java.util.List<XmlSuite>` | `getChildSuites()` |
| `java.lang.String` | `getConfigFailurePolicy()`             Returns the configuration failure policy. |
| `int` | `getDataProviderThreadCount()` |
| `java.util.List<java.lang.String>` | `getExcludedGroups()` |
| `java.lang.String` | `getFileName()` |
| `java.lang.Boolean` | `getGroupByInstances()` |
| `java.util.List<java.lang.String>` | `getIncludedGroups()` |
| `java.util.List<java.lang.String>` | `getListeners()` |
| `java.util.List<XmlMethodSelector>` | `getMethodSelectors()`             Returns the method selectors. |
| `java.lang.String` | `getName()`             Returns the name. |
| `ITestObjectFactory` | `getObjectFactory()` |
| `java.util.List<XmlPackage>` | `getPackages()` |
| `java.lang.String` | `getParallel()`             Returns the parallel mode. |
| `java.lang.String` | `getParameter(java.lang.String name)`             Returns the parameter defined in this suite only. |
| `java.util.Map<java.lang.String,java.lang.String>` | `getParameters()`             Gets the parameters that apply to tests in this suite.  Set of parameters for a suite is appended with parameters from parent suite. |
| `XmlSuite` | `getParentSuite()` |
| `java.lang.String` | `getPreserveOrder()` |
| `java.util.List<java.lang.String>` | `getSuiteFiles()`             Returns the suite files. |
| `java.lang.String` | `getTest()`             Returns the test. |
| `java.util.List<XmlTest>` | `getTests()`             Returns the tests. |
| `int` | `getThreadCount()`             Returns the threadCount. |
| `java.lang.String` | `getTimeOut()`             Returns the timeout. |
| `long` | `getTimeOut(long def)`             Returns the timeout as a long value specifying the default value to be used if no timeout was specified. |
| `java.lang.Integer` | `getVerbose()`             Returns the verbose. |
| `java.util.List<XmlPackage>` | `getXmlPackages()`             Returns the XML packages. |
| `int` | `hashCode()` |
| `java.lang.Boolean` | `isJUnit()`             Returns the JUnit compatibility flag. |
| `void` | `setAllowReturnValues(java.lang.Boolean allowReturnValues)` |
| `void` | `setConfigFailurePolicy(java.lang.String configFailurePolicy)`             Sets the configuration failure policy. |
| `void` | `setDataProviderThreadCount(int count)` |
| `void` | `setExcludedGroups(java.util.List<java.lang.String> g)` |
| `void` | `setFileName(java.lang.String fileName)` |
| `void` | `setGroupByInstances(boolean f)` |
| `void` | `setIncludedGroups(java.util.List<java.lang.String> g)` |
| `void` | `setJunit(java.lang.Boolean j)` |
| `void` | `setJUnit(java.lang.Boolean isJUnit)`             Sets the JUnit compatibility flag. |
| `void` | `setListeners(java.util.List<java.lang.String> listeners)` |
| `void` | `setMethodSelectors(java.util.List<XmlMethodSelector> methodSelectors)`             Sets the method selectors. |
| `void` | `setName(java.lang.String name)`             Sets the name. |
| `void` | `setObjectFactory(ITestObjectFactory objectFactory)` |
| `void` | `setPackages(java.util.List<XmlPackage> packages)` |
| `void` | `setParallel(java.lang.String parallel)`             Sets the parallel mode |
| `void` | `setParameters(java.util.Map<java.lang.String,java.lang.String> parameters)`             Sets parameters. |
| `void` | `setParentSuite(XmlSuite parentSuite)` |
| `void` | `setPreserveOrder(java.lang.String f)` |
| `void` | `setSkipFailedInvocationCounts(boolean skip)` |
| `void` | `setSuiteFiles(java.util.List<java.lang.String> files)`             Sets the suite files. |
| `void` | `setTests(java.util.List<XmlTest> tests)` |
| `void` | `setThreadCount(int threadCount)`             Set the thread count. |
| `void` | `setTimeOut(java.lang.String timeOut)`             Sets the timeout. |
| `void` | `setVerbose(java.lang.Integer verbose)`             Set the verbose. |
| `void` | `setXmlPackages(java.util.List<XmlPackage> packages)`             Sets the XML packages. |
| `java.lang.Boolean` | `skipFailedInvocationCounts()` |
| `java.lang.String` | `toString()` |
| `java.lang.String` | `toXml()`             Returns a String representation of this XML suite. |

**Methods inherited from class java.lang.Object**

`finalize, getClass, notify, notifyAll, wait, wait, wait`

**Field Detail**

### PARALLEL\_TESTS

```
public static final java.lang.String PARALLEL_TESTS
```

:   Parallel modes

    **See Also:**: Constant Field Values

---


### PARALLEL\_METHODS

```
public static final java.lang.String PARALLEL_METHODS
```

**See Also:**: Constant Field Values

---


### PARALLEL\_CLASSES

```
public static final java.lang.String PARALLEL_CLASSES
```

**See Also:**: Constant Field Values

---


### PARALLEL\_INSTANCES

```
public static final java.lang.String PARALLEL_INSTANCES
```

**See Also:**: Constant Field Values

---


### PARALLEL\_NONE

```
public static final java.lang.String PARALLEL_NONE
```

**See Also:**: Constant Field Values

---


### PARALLEL\_MODES

```
public static java.util.Set<java.lang.String> PARALLEL_MODES
```

---


### SKIP

```
public static final java.lang.String SKIP
```

:   Configuration failure policy options

    **See Also:**: Constant Field Values

---


### CONTINUE

```
public static final java.lang.String CONTINUE
```

**See Also:**: Constant Field Values

---


### DEFAULT\_VERBOSE

```
public static java.lang.Integer DEFAULT_VERBOSE
```

:   The suite verbose flag. (0 to 10)

---


### DEFAULT\_PARALLEL

```
public static java.lang.String DEFAULT_PARALLEL
```

---


### DEFAULT\_CONFIG\_FAILURE\_POLICY

```
public static java.lang.String DEFAULT_CONFIG_FAILURE_POLICY
```

:   Whether to SKIP or CONTINUE to re-attempt failed configuration methods.

---


### DEFAULT\_JUNIT

```
public static java.lang.Boolean DEFAULT_JUNIT
```

:   JUnit compatibility flag.

---


### DEFAULT\_MIXED

```
public static java.lang.Boolean DEFAULT_MIXED
```

:   mixed mode flag.

---


### DEFAULT\_SKIP\_FAILED\_INVOCATION\_COUNTS

```
public static java.lang.Boolean DEFAULT_SKIP_FAILED_INVOCATION_COUNTS
```

---


### DEFAULT\_THREAD\_COUNT

```
public static java.lang.Integer DEFAULT_THREAD_COUNT
```

:   The thread count.

---


### DEFAULT\_DATA\_PROVIDER\_THREAD\_COUNT

```
public static final java.lang.Integer DEFAULT_DATA_PROVIDER_THREAD_COUNT
```

:   Thread count for the data provider pool

---


### DEFAULT\_GROUP\_BY\_INSTANCES

```
public static final java.lang.Boolean DEFAULT_GROUP_BY_INSTANCES
```

:   By default, a method failing will cause all instances of that class to skip

---


### DEFAULT\_ALLOW\_RETURN\_VALUES

```
public static java.lang.Boolean DEFAULT_ALLOW_RETURN_VALUES
```

---


### DEFAULT\_PRESERVE\_ORDER

```
public static java.lang.String DEFAULT_PRESERVE_ORDER
```


**Constructor Detail**

### XmlSuite

```
public XmlSuite()
```


**Method Detail**

### getFileName

```
public java.lang.String getFileName()
```

:   **Returns:**: the fileName

---


### setFileName

```
public void setFileName(java.lang.String fileName)
```

:   **Parameters:**: `fileName` - the fileName to set

---


### getParallel

```
public java.lang.String getParallel()
```

:   Returns the parallel mode.

    :   **Returns:**: the parallel mode.

---


### getObjectFactory

```
public ITestObjectFactory getObjectFactory()
```

---


### setObjectFactory

```
public void setObjectFactory(ITestObjectFactory objectFactory)
```

---


### setParallel

```
public void setParallel(java.lang.String parallel)
```

:   Sets the parallel mode

    :   **Parameters:**: `parallel` - the parallel mode

---


### setConfigFailurePolicy

```
public void setConfigFailurePolicy(java.lang.String configFailurePolicy)
```

:   Sets the configuration failure policy.

    :   **Parameters:**: `configFailurePolicy` - the config failure policy

---


### getConfigFailurePolicy

```
public java.lang.String getConfigFailurePolicy()
```

:   Returns the configuration failure policy.

    :   **Returns:**: the configuration failure policy

---


### getVerbose

```
public java.lang.Integer getVerbose()
```

:   Returns the verbose.

    :   **Returns:**: the verbose.

---


### setVerbose

```
public void setVerbose(java.lang.Integer verbose)
```

:   Set the verbose.

    :   **Parameters:**: `verbose` - The verbose to set.

---


### getName

```
public java.lang.String getName()
```

:   Returns the name.

    :   **Returns:**: the name.

---


### setName

```
public void setName(java.lang.String name)
```

:   Sets the name.

    :   **Parameters:**: `name` - The name to set.

---


### getTest

```
public java.lang.String getTest()
```

:   Returns the test.

    :   **Returns:**: the test.

---


### getTests

```
public java.util.List<XmlTest> getTests()
```

:   Returns the tests.

    :   **Returns:**: the tests.

---


### setTests

```
public void setTests(java.util.List<XmlTest> tests)
```

---


### getMethodSelectors

```
public java.util.List<XmlMethodSelector> getMethodSelectors()
```

:   Returns the method selectors.

    :   **Returns:**: the method selectors.

---


### setMethodSelectors

```
public void setMethodSelectors(java.util.List<XmlMethodSelector> methodSelectors)
```

:   Sets the method selectors.

    :   **Parameters:**: `methodSelectors` - the method selectors.

---


### setParameters

```
public void setParameters(java.util.Map<java.lang.String,java.lang.String> parameters)
```

:   Sets parameters.

    :   **Parameters:**: `parameters` - the parameters.

---


### getParameters

```
public java.util.Map<java.lang.String,java.lang.String> getParameters()
```

:   Gets the parameters that apply to tests in this suite.  
    Set of parameters for a suite is appended with parameters from parent suite.
    Also, parameters from this suite override the same named parameters from
    parent suite.

---


### getAllParameters

```
public java.util.Map<java.lang.String,java.lang.String> getAllParameters()
```

:   **Returns:**: The parameters defined in this suite and all its XmlTests.

---


### getParameter

```
public java.lang.String getParameter(java.lang.String name)
```

:   Returns the parameter defined in this suite only.

    :   **Parameters:**: `name` - the parameter name. **Returns:**: The parameter defined in this suite only.

---


### getThreadCount

```
public int getThreadCount()
```

:   Returns the threadCount.

    :   **Returns:**: the threadCount.

---


### setThreadCount

```
public void setThreadCount(int threadCount)
```

:   Set the thread count.

    :   **Parameters:**: `threadCount` - The thread count to set.

---


### isJUnit

```
public java.lang.Boolean isJUnit()
```

:   Returns the JUnit compatibility flag.

    :   **Returns:**: the JUnit compatibility flag.

---


### setJUnit

```
public void setJUnit(java.lang.Boolean isJUnit)
```

:   Sets the JUnit compatibility flag.

    :   **Parameters:**: `isJUnit` - the JUnit compatibility flag.

---


### setJunit

```
public void setJunit(java.lang.Boolean j)
```

---


### skipFailedInvocationCounts

```
public java.lang.Boolean skipFailedInvocationCounts()
```

---


### setSkipFailedInvocationCounts

```
public void setSkipFailedInvocationCounts(boolean skip)
```

---


### setXmlPackages

```
public void setXmlPackages(java.util.List<XmlPackage> packages)
```

:   Sets the XML packages.

    :   **Parameters:**: `packages` - the XML packages.

---


### getXmlPackages

```
public java.util.List<XmlPackage> getXmlPackages()
```

:   Returns the XML packages.

    :   **Returns:**: the XML packages.

---


### getPackages

```
public java.util.List<XmlPackage> getPackages()
```

---


### setPackages

```
public void setPackages(java.util.List<XmlPackage> packages)
```

---


### toXml

```
public java.lang.String toXml()
```

:   Returns a String representation of this XML suite.

    :   **Returns:**: a String representation of this XML suite.

---


### toString

```
public java.lang.String toString()
```

:   :   **Overrides:**: `toString` in class `java.lang.Object`

---


### clone

```
public java.lang.Object clone()
```

:   Note that this is not a full clone: XmlTest children are not cloned by this
    method.

    :   **Overrides:**: `clone` in class `java.lang.Object`

---


### setTimeOut

```
public void setTimeOut(java.lang.String timeOut)
```

:   Sets the timeout.

    :   **Parameters:**: `timeOut` - the timeout.

---


### getTimeOut

```
public java.lang.String getTimeOut()
```

:   Returns the timeout.

    :   **Returns:**: the timeout.

---


### getTimeOut

```
public long getTimeOut(long def)
```

:   Returns the timeout as a long value specifying the default value to be used if
    no timeout was specified.

    :   **Parameters:**: `def` - the the default value to be used if no timeout was specified. **Returns:**: the timeout as a long value specifying the default value to be used if no timeout was specified.

---


### setSuiteFiles

```
public void setSuiteFiles(java.util.List<java.lang.String> files)
```

:   Sets the suite files.

    :   **Parameters:**: `files` - the suite files.

---


### getSuiteFiles

```
public java.util.List<java.lang.String> getSuiteFiles()
```

:   Returns the suite files.

    :   **Returns:**: the suite files.

---


### setListeners

```
public void setListeners(java.util.List<java.lang.String> listeners)
```

---


### getListeners

```
public java.util.List<java.lang.String> getListeners()
```

---


### setDataProviderThreadCount

```
public void setDataProviderThreadCount(int count)
```

---


### getDataProviderThreadCount

```
public int getDataProviderThreadCount()
```

---


### setParentSuite

```
public void setParentSuite(XmlSuite parentSuite)
```

---


### getParentSuite

```
public XmlSuite getParentSuite()
```

---


### getChildSuites

```
public java.util.List<XmlSuite> getChildSuites()
```

---


### hashCode

```
public int hashCode()
```

:   **Overrides:**: `hashCode` in class `java.lang.Object`

---


### equals

```
public boolean equals(java.lang.Object obj)
```

:   **Overrides:**: `equals` in class `java.lang.Object`

---


### setPreserveOrder

```
public void setPreserveOrder(java.lang.String f)
```

---


### getPreserveOrder

```
public java.lang.String getPreserveOrder()
```

---


### getIncludedGroups

```
public java.util.List<java.lang.String> getIncludedGroups()
```

:   **Returns:**: Returns the includedGroups. Note: do not modify the returned value, use `addIncludedGroup(String)`.

---


### addIncludedGroup

```
public void addIncludedGroup(java.lang.String g)
```

---


### setIncludedGroups

```
public void setIncludedGroups(java.util.List<java.lang.String> g)
```

:   **Parameters:**: `g` -

---


### setExcludedGroups

```
public void setExcludedGroups(java.util.List<java.lang.String> g)
```

:   **Parameters:**: `g` - The excludedGrousps to set.

---


### getExcludedGroups

```
public java.util.List<java.lang.String> getExcludedGroups()
```

:   **Returns:**: Returns the excludedGroups. Note: do not modify the returned value, use `addExcludedGroup(String)`.

---


### addExcludedGroup

```
public void addExcludedGroup(java.lang.String g)
```

---


### getGroupByInstances

```
public java.lang.Boolean getGroupByInstances()
```

---


### setGroupByInstances

```
public void setGroupByInstances(boolean f)
```

---


### addListener

```
public void addListener(java.lang.String listener)
```

---


### getAllowReturnValues

```
public java.lang.Boolean getAllowReturnValues()
```

---


### setAllowReturnValues

```
public void setAllowReturnValues(java.lang.Boolean allowReturnValues)
```


---


|  |  |  |
| --- | --- | --- |
| **Overview** \| **Package** \| **Class** \| **Tree** \| **Deprecated** \| **Index** \| **Help** | |  |
| **PREV CLASS**   **NEXT CLASS** | **FRAMES**    **NO FRAMES**     **All Classes** |  |
| SUMMARY: NESTED \| FIELD \| CONSTR \| METHOD | DETAIL: FIELD \| CONSTR \| METHOD |  |


---
